# Long-term survival of patients with thyroid cancer according to the methods of tumor detection: A nationwide cohort study in Korea

**DOI:** 10.1371/journal.pone.0194743

**Published:** 2018-04-16

**Authors:** Yuh-Seog Jung, Chang-Mo Oh, Yeol Kim, Kyu-Won Jung, Junsun Ryu, Young-Joo Won

**Affiliations:** 1 Head & Neck Oncology Clinic, Center for Thyroid Cancer, National Cancer Center Hospital, Goyang, South Korea; 2 Specific Organs Cancer Branch, Research Institute, National Cancer Center, Goyang, South Korea; 3 Department of Cancer Control and Population Health, National Cancer Center Graduate School of Cancer Science and Policy, National Cancer Center, Goyang, South Korea; 4 Cancer registration and statistic branch, National Cancer Control Institute, National Cancer Center, Goyang, South Korea; 5 Departments of Preventive Medicine, School of Medicine, Kyung Hee University, Seoul, South Korea; 6 Division of Cancer Management & Policy, National Cancer Control Institute, National Cancer Center, Goyang, South Korea; Institute of Experimental Endocrinology and Oncology (IEOS), ITALY

## Abstract

In this retrospective cohort study, we compared the survival of patients detected by screening with those detected based on symptoms, according to their tumor stages. After propensity score matching, 2,130 patients with papillary or follicular thyroid cancer, identified by screening detection (SD) and clinical detection (CD), were included. We compared the survival rates of patients identified by SD and CD in the early and advanced stages of thyroid cancer. Cox proportional hazard models were used to compare the hazard ratios (HRs) for mortality between the two groups. Of the 1,065 patients in each group, 12 (1.1%) died in the SD group, compared to 44 (4.1%) in the CD group, during an average 9.4 years (*p*<0.001). For early stage, there was no significant difference in all-cause and thyroid cancer-specific mortality between the two groups (*p* = 0.08, *p* = 0.0502). However, for advanced stage, the survival rates in the SD group were significantly higher than in the CD group (*p*<0.001, *p* = 0.004). Moreover, after adjusting for covariates, the HRs of all-cause mortality of the SD group was significantly lower than that of the CD group for the advanced stage patients (HRs: 0.37 [95% CIs: 0.17–0.80]), while no significant difference was observed in the early stage. While screening for thyroid cancer was not beneficial for early stage patients, our findings suggest that detection via screening is associated with better survival for patients with advanced stage cancer. However, the effects of selection bias and lead time bias could not be entirely excluded.

## Introduction

Over the past two decades, the incidence of papillary thyroid carcinoma has substantially increased worldwide [1,2]. Similarly, South Korea has witnessed a dramatic increase in the incidence of thyroid cancer [3]. In 2012, the age-standardized incidence rate of thyroid cancer was 62.5 per 100,000 in South Korea [4]. Despite this rapid increase in thyroid cancer incidence, the age-standardized mortality rate has remained stable [4]. Increased use of ultrasonography as an “add-on” during other cancer screening procedures has been considered to play a role in this steep increase [5,6]. Some researchers argued that this increase was just a “radiologic serendipity” of the indolent lesions that would not be otherwise clinically apparent during the patient’s lifetime. In Korea, this notion has led public arguments proposing that screening with ultrasonography be discouraged since early 2014 [5,7].

Despite the recent debates, evidence evaluating the real benefit or harm of thyroid cancer screening for the general population is still insufficient [8,9,10]. The U.S. Preventive Services Task Force (USPSTF) did not recommend thyroid cancer screening either by palpation or ultrasonography due to the lack of evidence both in 1996 [8], and more recently in 2017 [9]. In Korea, a government-led task force established the Guideline for Thyroid Cancer Screening, stating that “thyroid ultrasonography is not routinely recommended for healthy subjects because the gain or harm is not clearly defined at the current evidence level” in 2015 [10]. These guidelines reported that there were insufficient studies that evaluated the benefits and harms of routine thyroid cancer screening.

The Korean Central Cancer Registry (KCCR) conducted the National Epidemiologic Survey of Thyroid cancer (NEST), which was originally designed to collect data on the method of initial detection of cancer, and eventually to collect long-term survival data [11,12]. Particularly, a simple observational comparison of screening detection (SD) using ultrasonography versus clinical detection (CD), with long-term survival as a primary endpoint, is feasible using this dataset. However, the observational study is vulnerable to the lead time bias and length bias [13]. Thus herein, the survival rates for thyroid cancer patients were investigated by tumor stage to reduce the effects of lead time bias [13,14], and the study participants were restricted to only patients with well-differentiated thyroid cancer to reduce the effects of length bias.

Using the NEST dataset, we compared the all-cause and thyroid cancer-specific survival in thyroid cancer patients diagnosed by SD and by CD during an average of 9.4 follow-up years according to their tumor stages.

## Materials and methods

### Study design and participants

The NEST study was designed to collect representative samples of thyroid cancer patients diagnosed in the years 1999, 2005, and 2008 using a proportionally stratified and systematic random sampling method from the Korea National Cancer Incidence Database (KNCI DB) [11,12]. The routes of tumor detection were classified as SD (through cancer screening as recorded in medical records) and CD (by investigation of symptoms associated with thyroid disease, including thyroid cancer) [12].

The number of thyroid cancer patients registered in the KNCI DB was 3,342 in 1999, 12,659 in 2005, and 26,890 in 2008. We selected the study population using a two-stage sampling method for a given year. First, hospitals were randomly selected using a probability proportional to the size method stratified by regions in a given year. Different proportions of thyroid cancer patients were sampled for different years of study; 33% in 1999 (n = 1,103 patients), 22% in 2005 (n = 2,785 patients), and 11% in 2008 (n = 2,958). Of these sampled patients, 1,050 cases were excluded from the final analysis due to missing or inadequate variable data (n = 90 cases) and the refusal of two hospitals (n = 960 cases) among 24 hospitals. The NEST study data were linked to the cause of death database of the National Statistics Office in Korea [15]. The retrospective cohort study was conducted to compare the survival rates between the CD and SD groups. The cause of death data were used to define deaths from thyroid cancer. The ICD-10 code for death from thyroid cancer was “C73.” The research protocol for the present study was approved by the institutional review board of the National Cancer Center (IRB No: NCC2017-0070). Informed consent was not required because all data were fully anonymized before accessed. The NEST data is publicly opened and freely available.

### Case selection and propensity score matching

To match the basic characteristics of the SD and CD groups, we used the propensity score matching. A multiple logistic regression model was used to estimate the propensity score for the CD and SD groups after adjusting for age, sex, year of cancer diagnosis, and histologic types. A 1:1 matching was conducted with a caliper of 0.05 using the SAS macro program (% PS Matching) [16].

Of the 5,796 patients included in the NEST study, 5,672 patients who had well-differentiated thyroid cancer (papillary thyroid cancer, follicular thyroid cancer) were included in our study [17] because we thought that the restriction of study participants to only patients with well-differentiated thyroid cancer could reduce the effects of length bias.

**Fig 1:**
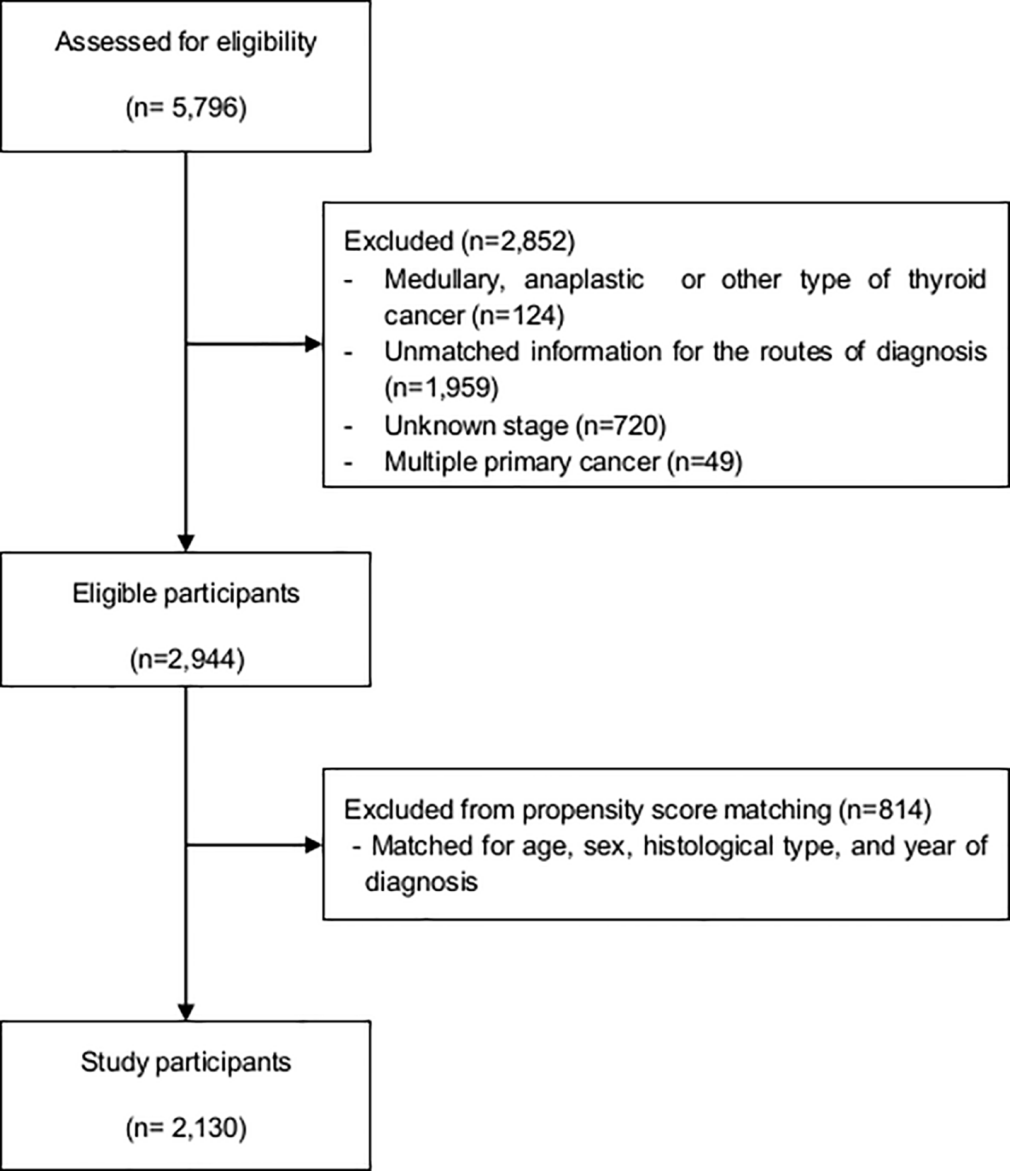
Flow-chart for selection of study participants.

Patients with unclear modes of tumor detection (n = 1959), unspecified stages of thyroid cancer (n = 720) and multiple primary tumors (n = 49) were all excluded from the study. After propensity score matching, 814 patients who did not have matched pairs were also excluded from our study. In the final analysis, 2,130 patients were included, and they were followed-up to the date of death or the closing date for the follow-up (December 31, 2015) (Fig 1).

### Statistical analysis

AJCC 6^th^ stage was used to classify the stage of thyroid cancer [18], because NEST study was conducted in year 2010 and stage of tumor also was collected in year 2010. The t-test and χ2-test were used to compare the differences in baseline characteristics of study participants between the SD and CD groups. The proportion of all-cause death and thyroid cancer-specific death was compared between the SD and CD groups after considering the TNM stage [18], lymph node involvement, distant metastasis, N1b involvement, extrathyroidal extension. The survival duration for patients with thyroid cancer was calculated as the time from the date of initial thyroid cancer diagnosis to the date of death or end of follow-up (December 31, 2015).

Since the SD group patients were more likely to have smaller tumors, which usually exhibit excellent survival and thus could lead to a bias in the calculation of mortality rate, analysis was performed by dividing study participants into two groups: early stage (stages I & II) and advanced stage (stages III & IV), according to the AJCC cancer staging manual guidelines [19].

The observed cumulative mortality rates determined by the Kaplan-Meier method were used to compare all-cause and thyroid cancer-specific mortality between the two groups in both the early stage and advanced stage patients. Log-rank tests were used to assess the differences between the Kaplan-Meier curves.

We also estimated the all-cause and thyroid cancer-specific mortality rate per 100,000 person-years for both the early stage and advanced stage groups. The 95% confidence intervals (CIs) for mortality rates were calculated per 100,000 people using the Poisson method. The Cox proportional hazard models were used to estimate adjusted hazard ratios (HRs) and their 95% CIs for all-cause and thyroid cancer-specific mortality after adjusting for age, sex and treatment method (Total thyroidectomy vs. less than total thyroidectomy vs. No surgery or unknown treatment method). *P* values less than 0.05 were considered statistically significant. All statistical analyses were performed using Stata 12.0 (Stata Corp LP, TX, U.S.A.) and SAS 9.2 (SAS Institute, Cary, NC, U.S.A.).

## Results

### Baseline characteristics of the screening and clinical detection groups

As shown in Table 1, the mean follow-up period was 9.4 years (range: 0.1~16.9 years) for both SD and CD groups. There were no significant differences in age, sex, and histologic type between the two groups. However, the number of overall and thyroid cancer-specific death cases was significantly different between the SD and CD groups. Although there was no significant difference in the incidences of distant metastasis between the two groups (*p* = 0.09), the CD group had more patients with advanced stage (*p*<0.001), lymph node metastasis (*p* = 0.03), N1b involvement (*p*<0.001), and extrathyroidal extension (*p* = 0.008).

**Table 1:** Comparison of the baseline characteristics of the screening and clinical detection groups.

Variables	Total	Diagnostic method		*P*-value
		Screening detection	Clinical detection	
Overall (n)	2,130	1,065	1,065	
Number of overall death	56 (2.6)	12 (1.1)	44 (4,1)	<0.001^†^
Number of death from thyroid cancer	25 (1.2)	3 (0.3)	22 (2.1)	<0.001^†^
Age (year)*	45.0 ± 12.2	44.5 ± 11.2	45.3 ± 13.2	0.16
	(13–86)	(15–86)	(13–79)	
Tumor size (mm) *	12.8 ± 10.9	10.3 ± 7.5	15.2 ± 13.0	<0.001^†^
		(n = 1,056)	(n = 1,051)	
Sex				
Men	248 (11.6)	123 (11.6)	125 (11.7)	0.89^†^
Women	1,882 (88.4)	942 (88.5)	940 (88.3)	
Diagnosed year				
1999 year	110 (5.2)	55 (5.2)	55 (5.2)	0.90
2005 year	1,150 (54.0)	570 (53.5)	580 (54.5)	
2008 year	870 (40.8)	440 (41.3)	430 (40.4)	
Histological type				
Follicular carcinoma	45 (2.1)	25 (2.4)	20 (1.9)	0.45^†^
Papillary carcinoma	2,085 (97.9)	1,040 (97.6)	1,045 (98.1)	
TNM stage				
Stage I	1,465 (68.8)	772 (72.5)	693 (65.1)	<0.001^†^
Stage II	17 (0.8)	9 (0.9)	8 (0.8)	
Stage III	458 (21.5)	217 (20.4)	241 (22.6)	
Stage IV	190 (8.9)	67 (6.3)	123 (11.6)	
Treatment (Surgery)				0.02^†^
Total thyroidectomy	1,739 (81.6)	887 (83.3)	852 (80.0)	
Less than total thyroidectomy	355 (16.7)	167 (15.7)	188 (17.7)	
No surgery or unknown	36 (1.7)	11 (1.0)	25 (2.4)	
Lymph node involvement				0.03^†^
Yes	780 (36.6)	361 (33.9)	419 (39.3)	
No	1,164 (54.7)	609 (57.2)	555 (52.1)	
Unknown	186 (8.7)	95 (8.9)	91 (8.5)	
Distant metastasis				0.09^†^
Yes	8 (0.4)	1 (0.1)	7 (0.6)	
No	2,119 (99.5)	1,062 (99.7)	1,057 (99.3)	
Unknown	3 (0.1)	2 (0.2)	1 (0.1)	
N1b				<0.001^†^
Yes	243 (11.4)	83 (7.8)	160 (15.0)	
No	1,701 (79.9)	887 (83.3)	814 (76.4)	
Unknown	186 (8.7)	95 (8.9)	91 (8.5)	
Extrathyroidal extension				0.008^†^
Yes	1,002 (47.0)	466 (43.8)	536 (50.3)	
No	1,096 (51.5)	584 (54.8)	512 (48.1)	
Unknown	32 (1.5)	15 (1.4)	17 (1.6)	

*Continuous variables are expressed as a mean ± standard deviation and the t-test was used to test differences between the screening and clinical detection groups.

^†^ Categorical variables are expressed as numbers (percentage), and a chi-square test was used to test the differences in proportions between the screening and clinical detection groups.

### Comparison of the proportions of all-cause deaths between the screening and clinical detection groups

In patients with stage IV thyroid cancer, the SD group (7.5%) had a lower proportion of all-cause death cases compared to the CD group (19.5%) (Table 2). However, in patients with stage I-II thyroid cancer, no significant difference was seen in the proportion of all-cause death cases between the SD and CD groups. Furthermore, regardless of the lymph node or N1b involvement, the SD group had a lower proportion of all-cause death cases than the CD group. While there was no significant difference in the proportion of all-cause death cases between the SD and CD groups in patients with distant metastasis (*p* = 0.99), it was significantly lower in the SD group in patients with extrathyroidal extension (*p* = 0.002).

**Table 2:** Comparison of the number of deaths between the screening and clinical detection groups.

Variables	Total	Diagnostic method		*P*-value*
		Screening detection (n = 1,065)	Clinical detection (n = 1,065)	
**All-cause death**				
Total	56/2,130 (2.6)	12/1,065 (1.1)	44/1,065 (4.1)	<0.001
Stage I	13/1,465 (0.9)	4/772 (0.5)	9/693 (1.3)	0.16
Stage II	1/17 (5.9)	0/9 (0.0)	1/8 (12.5)	0.47
Stage III	13/458 (2.8)	3/217 (1.4)	10/241 (4.2)	0.09
Stage IV	29/190 (15.3)	5/67 (7.5)	24/123 (19.5)	0.03
Lymph node involvement				
Yes	30/780 (3.9)	6/361 (1.7)	24/419 (5.7)	0.004
No	24/1,164 (2.1)	6/609 (1.0)	18/555 (3.2)	0.007
Unknown	2/186 (1.1)	0/95 (0.0)	2/91 (2.2)	0.99
Distant metastasis				
Yes	4/8 (50.0)	0/1 (0.0)	4/7 (57.1)	0.99
No	51/2,119 (2.4)	12/1,062 (1.1)	39/1,057 (3.7)	<0.001
Unknown	1/3 (33.3)	0/2 (0.0)	1/1 (100.0)	0.33
N1b involvement				
Yes	18/243 (7.4)	2/83 (2.4)	16/160 (10.0)	0.04
No	36/1,701 (2.1)	10/887 (1.1)	26/814 (3.2)	0.004
Unknown	2/186 (1.1)	0/95 (0.0)	2/91 (2.2)	0.24
Extrathyroid invasion				
Yes	35/1,002 (3.5)	7/466 (1.5)	28/536 (5.2)	0.002
No	15/1,096 (1,4)	5/584 (0.9)	10/512 (2.0)	0.13
Unknown	6/32 (18.8)	0/15 (0.0)	6/17 (35.3)	0.02
**Thyroid cancer-specific death**				
Total	25/2,130 (1.2)	3/1,065 (0.3)	22/1,065 (2.1)	<0.001
Stage I	4/1,465 (0.3)	0/772 (0.0)	4/693 (0.6)	0.049
Stage II	0/17 (0.0)	0/9 (0.0)	0/8 (0.0)	-
Stage III	3/458 (0.7)	0/217 (0.0)	3/241 (1.2)	0.25
Stage IV	18/190 (9.5)	3/67 (4.5)	15/123 (12.2)	0.19
Lymph node involvement				
Yes	15/780 (1.9)	2/361 (0.6)	13/419 (3.1)	0.02
No	8/1,164 (4.6)	1/609 (0.2)	7/555 (1.3)	0.03
Unknown	2/186 (1.1)	0/95 (0.0)	2/91 (2.2)	0.24
Distant metastasis				
Yes	4/8 (50.0)	0/1 (0.0)	4/7 (57.1)	0.99
No	20/2,119 (0.9)	3/1,062 (0.3)	17/1,057 (1.6)	0.001
Unknown	1/3 (33.3)	0/2 (0.0)	1/1 (100.0)	0.33
N1b involvement				
Yes	14/243 (5.8)	2/83 (2.4)	12/160 (7.5)	0.15
No	9/1,701 (0.5)	1/887 (0.1)	8/814 (1.0)	0.02
Unknown	2/186 (1.1)	0/95 (0.0)	2/91 (2.2)	0.24
Extrathyroid invasion				
Yes	15/1,002 (1.5)	3/466 (0.6)	12/536(2.2)	0.06
No	4/1,096 (0.4)	0/584 (0.0)	4/512 (0.8)	0.047
Unknown	6/32 (18.8)	0/15 (0.0)	6/17 (35.3)	0.02

*The Fisher’s exact test or chi-square test were used to test differences in the distribution of characteristics between the screening and clinical detection groups.

### Comparison of the proportions of thyroid cancer-specific deaths between the screening and clinical detection groups

Although the number of deaths cases from thyroid cancer was lower in the SD group (4.5%) than in the CD group (12.2%) among patients with stage IV thyroid cancer, the difference was not significant (*p* = 0.19) (Table 2). While there were four deaths from thyroid cancer among the CD group of patients with stage I cancer, there were none in the SD group of patients with stage I or II cancer. Moreover, in patients with worse prognostic factors (e.g., distant metastasis, N1b involvement, extrathyroid extension), the number of deaths cases from thyroid cancer was lower in the SD group than in the CD group. There were no significant differences in the proportion of deaths from thyroid cancer between the SD and CD groups among patients with distant metastasis, N1b involvement or extrathyroid extension.

### Cumulative mortality rates for all-cause deaths between the screening and clinical detection groups

**Fig 2:**
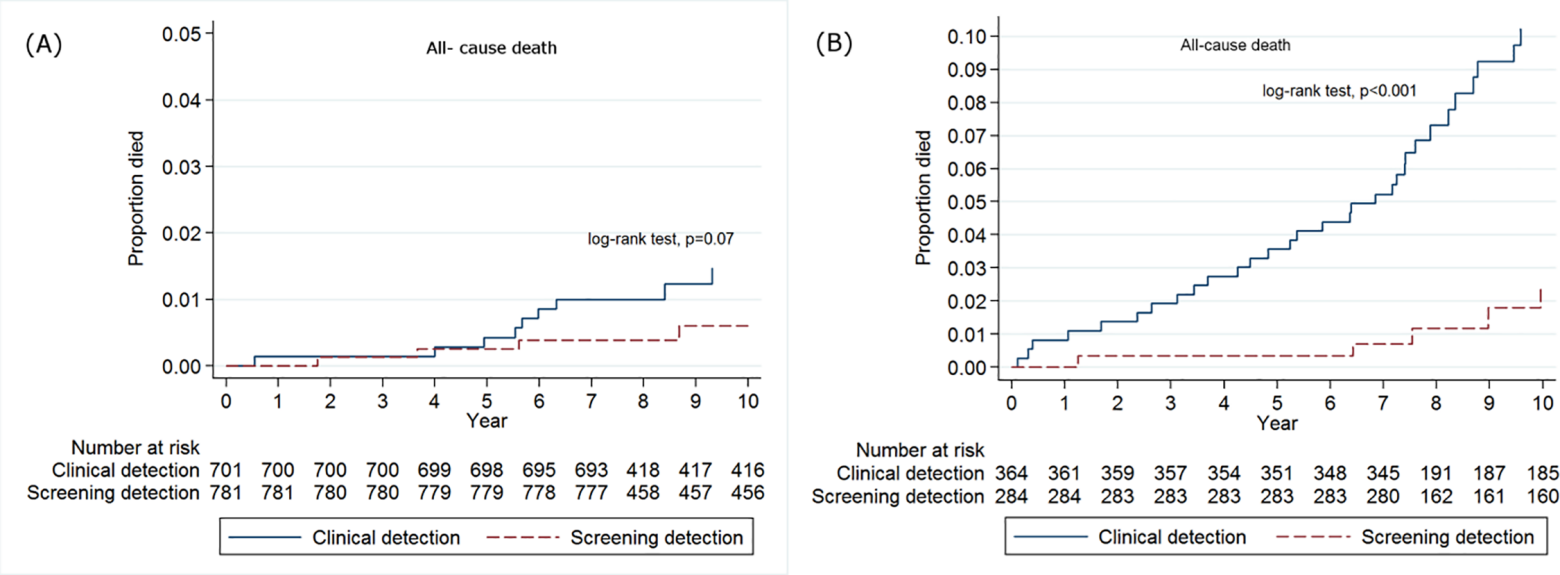
Kaplan–Meier plot of cumulative mortality for all-cause death between screening detection and clinical detection groups in patients. Footnotes: A. Patients with early stage thyroid cancer (stage I & II according to 6th edition of AJCC cancer staging manual) B. Patients with advanced stage thyroid cancer (stage III & IV according to 6th edition of AJCC cancer staging manual). *Log-rank tests were used to assess the differences in cumulative mortality between the clinical detection and the screening detection groups.

The cumulative mortality rates for all-cause deaths were compared between the two groups. There was no significant difference in all-cause deaths between the SD and CD groups among patients with stage I or II thyroid cancer (log-rank test, *p* = 0.07) (Fig 2A). However, cumulative mortality risk for all-cause deaths in the CD group were significantly higher than in the SD group among patients with stage III or IV thyroid cancer (log-rank test, *p*<0.001) (Fig 2B).

### Cumulative mortality rates for thyroid cancer-specific deaths between the screening and clinical detection groups

**Fig 3:**
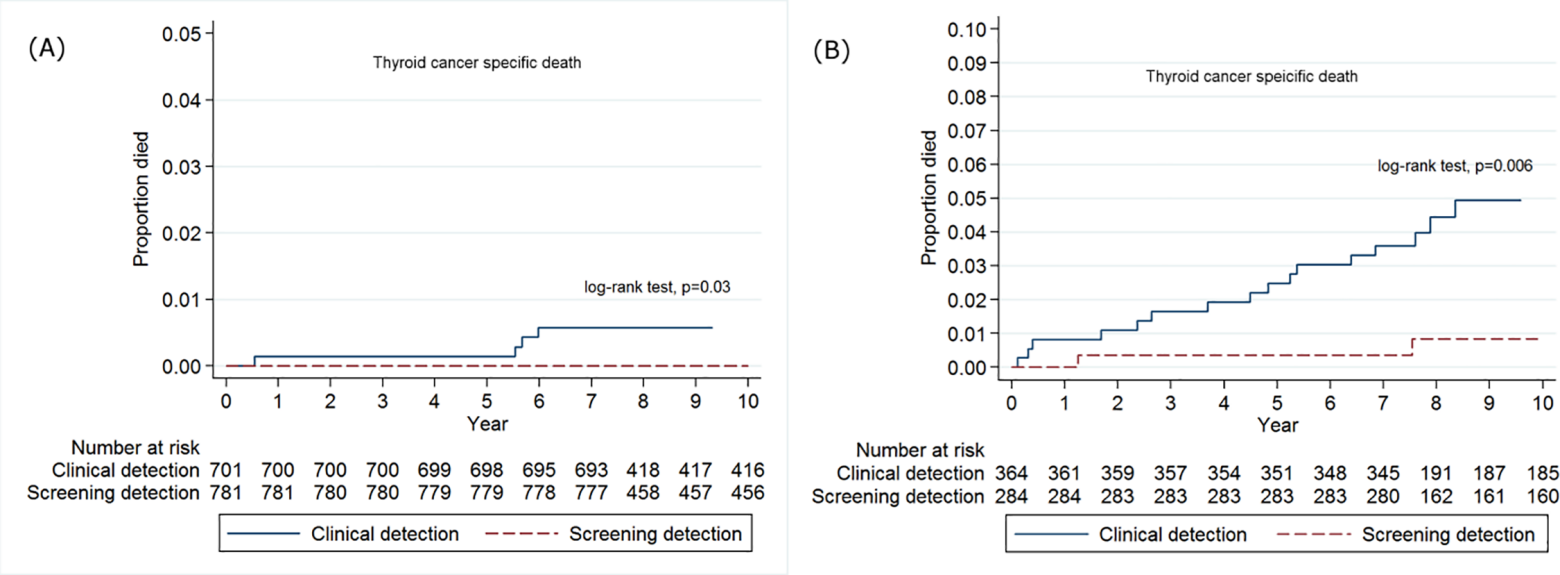
Kaplan–Meier plot of cumulative mortality for thyroid cancer-specific deaths between screening detection and clinical detection groups among patients. Footnotes: A. Patients with early stage thyroid cancer (stage I & II according to 6th edition of AJCC cancer staging manual) B. Patients with advanced stage thyroid cancer (stage III & IV according to 6th edition of AJCC cancer staging manual) *Log-rank tests were used to assess the differences in cumulative mortality between clinical detection and screening detection groups.

Among patients with stage I or II thyroid cancer, the cumulative mortality rate for thyroid cancer-specific deaths in the CD group was higher than in the SD group (log-rank test, *p* = 0.03); however, the probability of death from thyroid cancer for the CD group was less than 1% (Fig 3A). Furthermore, there were clear differences in the cumulative mortality rates for thyroid cancer-specific deaths between the SD and CD groups among patients with advanced stage thyroid cancer (log-rank test, *p* = 0.006) (Fig 3B).

### Hazard ratio for all-cause deaths between the screening and clinical detection groups

We compared the mortality risks for all-cause deaths between the SD and CD groups among patients with thyroid cancer (Table 3). In the fully adjusted Cox-proportional hazard model, there was no significant difference in the mortality risk between the SD and CD groups (p = 0.09) with early stage thyroid cancer (stage I & II, but the risk of all-cause mortality in the SD group was significantly lower (HR, 0.37 [95% CI, 0.17 to 0.80]) than in the CD group after adjusting for age, sex, and treatment method among patients with advanced stage thyroid cancer (stage III & IV).

**Table 3:** Comparison of all-cause mortality between the screening and clinical detection groups among patients with thyroid cancer.

	Mortality proportion	Mortality rate per 1,000 person-years (95% CIs)	Hazard ratios (95% CIs)	
			Unadjusted HRs	Adjusted HRs
**Stage I-II thyroid cancer**				
Clinical detection	10/701	1.50 (0.81 to 2.78)	1.00 (Reference)	1.00 (Reference)
Screening detection	4/781	0.54 (0.20 to 1.43)	0.36 (0.11 to 1.14)	0.36 (0.11 to 1.16)
**Stage III-IV thyroid cancer**				
Clinical detection	34/364	10.32 (7.37 to 14.44)	1.00 (Reference)	1.00 (Reference)
Screening detection	8/284	3.01 (1.50 to 6.01)	0.29 (0.14 to 0.63)	0.37 (0.17 to 0.80)

HRs = Hazard ratios; 95% CIs = 95% Confidence intervals

Mortality proportion was expressed as the number of death divided by the overall patients with stage Iaor II thyroid cancer.

Mortality rates were calculated as the number of deaths per 1,000 person-years.

Adjusted HR for all-cause and thyroid cancer-specific mortality were modeled after adjusting for age, sex and treatment method.

### Hazard ratio for thyroid cancer-specific deaths between the screening and clinical detection groups

Although the absolute number of deaths from thyroid cancer were very few in both groups, there was no significant difference in the thyroid cancer specific-mortality rate between the SD and CD groups among patients with early stage thyroid cancer (stage I & II (*p* = 0.0502). In the fully adjusted Cox-proportional hazards model, the hazard ratios for thyroid cancer-specific deaths among patients with advanced stage thyroid cancer (stage III & IV) were also lower in the SD group (HR, 0.27 [95% CI, 0.08 to 0.95]) compared to the CD group (Table 4).

**Table 4:** Comparison of thyroid cancer specific-mortality between the screening and clinical detection groups, among patients with thyroid cancer.

	Mortality proportion	Mortality rate per 1,000 person-years (95% CIs)	Hazard ratios (95% CIs)	
			Unadjusted HRs	Adjusted HRs
**Stage I-II thyroid cancer**				
Clinical detection	4/701	0.60 (0.22 to 1.59)	1.00 (Reference)	1.00 (Reference)
Screening detection	0/781	0.00 (0.00 to 0.00)	-	-
**Stage III-IV thyroid cancer**				
Clinical detection	18/364	5.46 (3.44 to 8.67)	1.00 (Reference)	1.00 (Reference)
Screening detection	3/284	1.13 (0.36 to 3.50)	0.21 (0.06 to 0.71)	0.27 (0.08 to 0.95)

HRs = Hazard ratios; 95% CIs = 95% Confidence intervals

Mortality proportion was expressed as the number of death divided by the overall patients with stage III aor IV thyroid cancer.

Mortality rates were calculated as the number of deaths per 1,000 person-years.

Adjusted HR for all-cause and thyroid cancer-specific mortality were modeled after adjusting for age, sex and treatment method.

## Discussion

The precise role of cancer screening techniques for reducing overall and cancer-related mortality should be evaluated through randomized controlled trials [13]. However, owing to the low mortality rate and a long latency, it might be difficult to find strong evidence, especially for well-differentiated thyroid cancer. In our study, we were able to observe the long-term prognosis of patients with thyroid cancer based on the methods of detection [12]. We found significant differences in the long-term mortality rates in patients with advanced stage thyroid cancer, but not in patients with early stage cancer. These findings suggest that screening for early stage thyroid cancer might not be beneficial.

Until now, very few reports have described the different characteristics of thyroid cancer based on the modes of detection, and the effectiveness of thyroid cancer screening is still uncertain. Only one cohort study in Japan showed that the cumulative survival rate of thyroid cancer patients detected by screening (98%) was higher than that of patients detected by symptoms (90%) [20]. However, the study in Japan was prone to lead time bias and length bias. There is no clear evidence of the down-staging effect of thyroid cancer screening yet. Choi et al. conducted a retrospective review and found that patients detected by screening had a higher percent of only microcarcinomas, but there was no difference in the overall stage and nodal metastasis [21]. Similarly, Chung et al. and Yamada et al. reported that stage, extracapsular spread, central and lateral lymphatic metastasis, and distant metastasis were not different in the SD cases [22,23]. However, in our study, more patients in the CD group had distant metastasis, which is the most important prognostic indicator for poor survival [24], as well as lymph node metastasis, especially N1b, which is a risk factor for local recurrence and even survival, although it was not significant [25].

Our data also showed that after matching for age, sex, year of diagnosis, and histological type, there were significant differences in the incidences of all-cause deaths and thyroid cancer-specific deaths between the CD and SD groups, among patients with advanced thyroid cancer. For example, the SD group showed lower proportions of all-cause and thyroid cancer-specific mortality than the CD group, even within the same stage IV thyroid cancer, disease with lymph node involvement, N1b, extrathyroid invasion, and distant metastasis, although the differences were not statistically significant.

One possible explanation for the improved survival in the SD group of patients with advanced thyroid cancer is the stage migration effect. Although we could not review the detailed clinical manifestations of these mortality cases, we speculate that SD helped in the detection of these advanced cases before their severe progression, and this eventually was associated with better survival rates in the SD group. The tumors in the SD group were more likely to have a lower number of metastases and a smaller metastatic size or extracapsular spread, as expected. Moreover, it is possible that SD group had less severe extrathyroid invasion and distant metastasis. Indeed, the age-standardized mortality rate for thyroid cancer has decreased steadily from 2004 to 2015 [15], with a lag time of the year 1999, during which ultrasound examination for the thyroid gland started to spread throughout South Korea [5,12].

Another possible explanation is that worse survival among the CD group patients with advanced stage thyroid cancer was due to disparities in the socioeconomic status [26] or co-morbidity [27]. In our study, the SD group had an overall lower all-cause mortality risk compared to the CD group. In addition, the 5-year relative survival rate for thyroid cancer was over 100% in South Korea [28]. This means that patients diagnosed with thyroid cancer might be healthier and had a lower risk of dying compared to the general population [11]. It is also possible that patients with advanced thyroid cancer who were detected by SD had better prognosis due to the selection of a better hospital or treatment option due to their higher income level.

As our data indicated, there may be no additional benefits of early screening for thyroid cancer. However, among patients with advanced stage cancer, the SD group had better survival rates than the CD group. Therefore, further studies will be required to establish whether the improved survival rates seen among advanced thyroid cancer patients detected via screening actually indicate a stage migration effect or are a mere result of the disparity in socioeconomic status or comorbidity between two groups. We also need to find ways of identifying populations that are at high risk for such clinical situations. The criteria to define the patients at high risk should be determined via clinical investigations. For example, a decision-tree model showed that thyroid cancer screening by ultrasonography was a cost-effective strategy for a high-risk subgroup among an obese population, although thyroid cancer screening for the general population was not cost-effective [29]. It is, therefore, necessary to develop a new personalized strategy to distinguish between high-risk patients who are likely to benefit from thyroid cancer screening and those with low risk for thyroid cancer progression [30]. Unfortunately, it was impossible to distinguish between potentially high-risk patients and low-risk patients in our study.

The main limitation of our analysis is that NEST dataset was not originally designed for a randomized controlled trial. Although we tried to minimize the potential bias through this analysis, lead time and length biases could not be fully removed, because the dataset is not a cohort of the normal population. However, we conducted stratified analysis by tumor stage. Therefore, it is likely to be prone to lead time bias or length bias in the early stage patients, but they would be less likely to affect our findings with the advanced stage patients. Other confounders that could influence the mortality, such as socioeconomic status, comorbidities, body mass index, smoking habits and regions, could not be adjusted using our dataset, which could potentially give a biased result. Indeed, there were significant differences in all-cause mortality between SD group and CD group; it may, in fact, underscore the presence of these confounding factors. Another limitation of our study is that there may be a bias in classifying the routes of detection. However, there was no significant difference in the estimated incidence rates for total thyroid cancer using NEST dataset compared to the nationally reported incidence rate of thyroid cancer [12]. Selection bias may be a critical point to interpret our results. More than half of participants were excluded due to missing or no match in the propensity score matching. Finally, we could not consider the potential harm of screening for thyroid cancer patients. Thyroid cancer screening by ultrasonography seems to have minimal potential harm, but detection of cancer is directly associated with treatment such as surgery or radioactive iodine. Overdiagnosis of thyroid cancer may lead to exposure to potential harm such as recurrent laryngeal nerve palsy or hypoparathyroidism.

In conclusion, the analysis of a nationally representative sample for thyroid cancer in Korea (NEST) showed that screening for thyroid cancer was associated with a reduction in overall and thyroid cancer-specific mortality in the advanced stage cancers, but not in the early stage cancers. Based on our findings, we do not recommend the routine use of thyroid cancer screening, especially among patients with early stage thyroid cancer; however, further studies are necessary to distinguish clinically-significant tumors from indolent, “non-lethal” thyroid cancers, and to verify whether personalized thyroid cancer screening is beneficial in improving survival among patients with advanced thyroid cancer.
